# HIV-1 Tat-induced VAPB disruption initiates a cascade of organellar failures culminating in neuronal lipid accumulation

**DOI:** 10.1016/j.jlr.2026.101053

**Published:** 2026-05-04

**Authors:** Maryline Santerre, Sterling P. Arjona, Kathy Q. Cai, Natalia Shcherbik, Bassel E. Sawaya

**Affiliations:** 1 FELS Cancer Institute for Personalized Medicine, Lewis Katz School of Medicine - Temple University, Philadelphia, PA, USA; 2 Histopathology Facility, Fox Chase Cancer Center, Philadelphia, PA, USA; 3 Department of Cell and Molecular Biology, School of Osteopathic Medicine, Rowan University, Stratford, NJ, USA; 4 Department of Cancer and Cellular Biology, Lewis Katz School of Medicine - Temple University, Philadelphia, PA, USA; 5 Department of Neural Sciences, Lewis Katz School of Medicine - Temple University, Philadelphia, PA, USA

**Keywords:** HIV-1 Tat, VAPB, triglycerides, lipid droplets, stroke, HAND, mitochondria-ER contacts

## Abstract

People living with HIV develop persistent neurocognitive impairment despite viral suppression through incompletely defined mechanisms. HIV-1 Tat disrupts VAPB-PTPIP51 coupling at mitochondria-associated ER membranes via PTPIP51 tyrosine phosphorylation, causing VAPB relocalization away from MAMs, a causal mechanism established in our prior work. Here, we define the downstream metabolic consequences and establish VAPB as the critical determinant of neuronal lipid pathology. Lipidomic profiling identified triglycerides as the dominant altered species, comprising polyunsaturated forms normally destined for membrane synthesis or mitochondrial oxidation, consistent with membrane catabolism rather than de novo lipogenesis. Targeted metabolomics revealed bioenergetic collapse consistent with impaired mitochondrial oxidative function. The resulting lipid imbalance, including lipid droplet accumulation, produced secondary organellar dysfunction, including Golgi dispersal and ER stress. Critically, Tat failed to induce lipid droplet accumulation in shRNA-VAPB cells, while PTPIP51 silencing had no such protective effect, establishing that VAPB relocalization is the obligate trigger. Guanosine supplementation reduced lipid droplet accumulation, suggesting a link to bioenergetic failure that warrants further investigation. In postmortem HIV-infected frontal cortex, VAPB was paradoxically elevated yet correlated with worsening dementia severity, consistent with transcriptional upregulation that cannot overcome posttranslational blockade of VAPB-MAM localization. The polyunsaturated triglycerides, depleted plasmalogens, and elevated ceramides documented here closely parallel lipid signatures reported in PLWH with cerebrovascular complications, implicating Tat-driven lipid dysregulation as a candidate mechanism for the incompletely explained elevation in stroke risk in this population.

People living with HIV face persistent neurocognitive impairment despite effective antiretroviral therapy and viral suppression. HIV-associated neurocognitive disorders affect 30%–50% of the treated individuals, with mechanisms incompletely understood (1, 2, 3, 4, 5). HIV-1 Tat protein persists in cerebrospinal fluid despite suppressive therapy and accumulates in neurons, where it induces calcium dysregulation, mitochondrial dysfunction, and ER stress (6, 7, 8, 9, 10). These cellular stresses converge on mitochondria-associated ER membranes (MAMs), which are contact sites that coordinate calcium signaling, lipid synthesis, and bioenergetics (11, 12).

VAPB is a tail-anchored ER protein that tethers ER to multiple organelles. VAPB-PTPIP51 complexes maintain MAM integrity, while VAPB-YIF1A interactions coordinate ER-Golgi trafficking (13, 14, 15, 16). VAPB mutations cause familial amyotrophic lateral sclerosis), and their dysfunction occurs in Parkinson's disease and Alzheimer's disease (14, 16, 17). The causal mechanism underlying VAPB disruption by Tat was established in our prior published study: Tat promotes PTPIP51 tyrosine phosphorylation, prevents PTPIP51-VAPB binding, and depletes both proteins from MAM fractions despite unchanged whole-cell expression (11). Critically, kinase inhibitor treatment restored PTPIP51-VAPB interaction and overcame the effects of Tat, providing direct experimental evidence of causality. The current manuscript builds on that established causal foundation to characterize the downstream metabolic consequences of VAPB disruption.

Lipid droplet accumulation in neurons is emerging as a hallmark of metabolic stress (18, 19, 20, 21, 22, 23, 24, 25). Unlike astrocytes and microglia, neurons lack robust lipid storage capacity and are exquisitely vulnerable to metabolic perturbations (22, 24, 26). Lipid droplets form in response to the same cellular stresses induced by Tat: calcium overload, mitochondrial dysfunction, and ER stress (18, 19, 20). Recent work has demonstrated Tat-induced lipid droplet formation in microglia via SREBP2-mediated lipogenesis (27, 28), but whether Tat induces neuronal lipid accumulation through similar mechanisms or through distinct pathways related to VAPB-dependent organellar dysfunction remains unexplored (29).

Here, we used comprehensive multi-omics profiling to examine whether VAPB disruption is associated with neuronal lipid accumulation. We found that Tat initiates a VAPB-related cascade in which disruption of VAPB-dependent organellar contacts occurs before ER stress (or, as we mentioned in the manuscript, Tat employs two distinct mechanisms to cause both events), resulting in proteostatic failure, lipid accumulation, and bioenergetic collapse. Loss-of-function experiments using shRNA identified VAPB as a key player, with VAPB silencing mimicking Tat effects, whereas PTPIP51 silencing did not. These results highlight VAPB as an important mechanistic candidate whose disruption relates to various aspects of HIV neuropathogenesis.

## Materials and methods

### Cell culture and transfection

SH-SY5Y neuroblastoma cells were purchased from ATCC (CRL-2266) and maintained in DMEM/F12 with 10% fetal bovine serum at 37°C with 5% CO_2_. Cells were differentiated with 10 μM retinoic acid for at least 4 days before treatment and subsequent experiments. Retinoic acid differentiation produces cells with neuronal morphology and reduced proliferation; however, we note that these remain a transformed cell line and may not fully recapitulate the physiology of terminally differentiated primary neurons. GFP-LC3 (Addgene, 24,920) and mCherry-LAMP (Addgene, 45,147) (30) expression plasmids were transfected using Lipofectamine. Controls included tunicamycin (5 μg/ml), oleic acid (30 μM, lipid droplet positive control, data not shown), and guanosine (100 μM).

### Tat treatment

Recombinant Tat protein was obtained from the NIH-AIDS Reagent Program (HIV-1 IIIB Tat Protein, ARP-2222). The protein was reconstituted according to the datasheet in PBS containing 1 mg/ml BSA and 0.1 mM DTT. All experimental cells were treated with 100 ng/ml Tat or with PBS containing BSA and DTT (labeled control) for 24 h, as indicated. Heat-inactivated Tat was prepared by boiling at 100 °C for 10 min.

### Western blot

Cells were lysed in RIPA buffer (25 mM Tris-HCl pH 7.6, 150 mM NaCl, 1% Triton X-100, 0.1% SDS). Primary antibodies: CHOP, BiP/GRP78, p-eIF2α(S51), total eIF2α, GOLGA2/GM130, and GAPDH (rabbit 14C10, Cell Signaling; and mouse D-6: sc-166545, Santa Cruz). Secondary antibodies: anti-mouse IgG-HRP (1:10,000; Advansta R-05071-500) and anti-rabbit IgG-HRP (1:10,000; Advansta R-05072-500). All blot images indicate molecular weight marker positions above and below bands of interest. Cropped images indicate splice sites with visible demarcation. Densitometry was performed using ImageJ with normalization to GAPDH loading control. Individual data points and SD are shown on all quantification graphs.

### Lipidomics and metabolomics

Cells were harvested 24 h post-treatment. For lipidomics, lipids were extracted from 11.6×106 cells per condition (n = 4 biological replicates) using the Bligh-Dyer method and analyzed by LC-MS/MS at the Wistar Institute. Data were normalized using the Splash method. For metabolomics, 6 × 10^6^ cells per condition (n = 3 replicates) were analyzed by LC-MS/MS with QCSum normalization. Significance: fold change greater than 1.5, *P* < 0.05 by unpaired *t* test.

### Microscopy and staining

Cells were fixed in 4% paraformaldehyde, permeabilized with 0.1% Triton X-100, and stained with BODIPY 493/503 (lipid droplets), anti-α-synuclein antibody (Cell Signaling), and DAPI (nuclei). For Oil Red O staining, cells were incubated in 60% isopropanol, then in Oil Red O solution for 10 min. Golgi morphology was assessed with anti-GOLGA2/GM130 (BD Biosciences). Images were acquired on a Leica EL6000 DMI3000 confocal microscope.

### Lipid droplet quantification

BODIPY-stained cells were imaged with acquisition settings maintained across all conditions. For each condition, at least 50 cells were analyzed across three independent experiments. Images were processed in ImageJ/Fiji using the Analyze Particles function (8 bit, rolling-ball background subtraction, radius 50 pixels, Otsu thresholding). Individual lipid droplets were identified as BODIPY-positive particles with areas greater than 0.1 μm^2^ and circularities greater than 0.5. Parameters measured: (1) number of lipid droplets per cell, (2) individual droplet area (μm^2^), and (3) total lipid droplet area per cell. Data are presented as mean ± SEM. Statistical comparisons: one-way ANOVA with Tukey's post-hoc test.

### VAPB knockdown

pLKO.1 puro with shRNA construct was obtained from Addgene. PTPIP51 and VAPB primers were purchased from and cloned into the shRNA construct as shown in Fig. 4A. PTPIP51: (forward) 5′-ccgggtgagcgagaagaagtcatatctcgagatatgacttcttctcgctcactttttg-3’; (reverse) 5′-aattcaaaaagtgagcgagaagaagtcatatctcgagatatgacttcttctcgctcac. VAPB: (forward) 5′-ccggccagttctgttt gactatgtactcgagtacatagtcaaacagaactggtttttg-3’; (reverse) 5′-aattcaaaaaccagttctgtttgactatgtactcgagt acatagtcaacatara ct. Lentiviral shRNA targeting VAPB or scrambled control (pLKO.1 vector) was transduced into cells, followed by puromycin selection. Knockdown was confirmed by Western blotting and immunofluorescence prior to Tat treatment.

### Human brain tissue

Postmortem frontal cortex from normal (n = 3), HIV-positive (n = 3), and HIV-positive with dementia (n = 3) was obtained from the National NeuroAIDS Tissue Consortium (NNTC). Paraffin sections underwent antigen retrieval and immunohistochemistry with anti-VAPB and anti-PTPIP51 antibodies. Staining intensity was quantified as the percentage of positive area using ImageJ color deconvolution.

### Statistical analysis

Data are mean ± SEM. Individual data points are shown on all bar graphs in accordance with the journal requirements. Comparisons used unpaired t-tests or Mann-Whitney tests after Shapiro-Wilk normality testing. Multiple group comparisons: one-way ANOVA with Tukey's post hoc test. Significance: *P* < 0.05.

### Human ethical statement

All experiments involving human brain tissue samples were conducted in accordance with the guidelines set by the National Institutes of Health (NIH). De-identified human tissue samples—limited to information on gender, age, and HIV status—were obtained from the National NeuroAIDS Tissue Consortium (NNTC) and approved by the Institutional Biosafety Committee (IBC) (IBC #11182, approved on 03/12/2024 – PI: Dr Bassel E Sawaya) and the Institutional Review Board (IRB) (protocol #4195, approved on 2/24/2025 – PI: Dr Bassel E Sawaya) at Temple University. Consent from guardians was not required because the samples were de-identified prior to receipt.

### Software and data analysis

Image quantification was performed using ImageJ (National Institutes of Health, Bethesda, MD; https://imagej.nih.gov/ij/). Statistical analyses were conducted using GraphPad Prism (GraphPad Software, San Diego, CA; https://www.graphpad.com). Metabolomics data were analyzed using MetaboAnalyst, developed by the Wishart Research Group at the University of Alberta (Edmonton, Alberta, Canada; https://www.metaboanalyst.ca/). Lipidomics data were analyzed using custom computational workflows for lipid-class aggregation and comparative profiling, with lipid annotation following LIPID MAPS nomenclature (https://www.lipidmaps.org/).

## Results

**Fig. 1:**
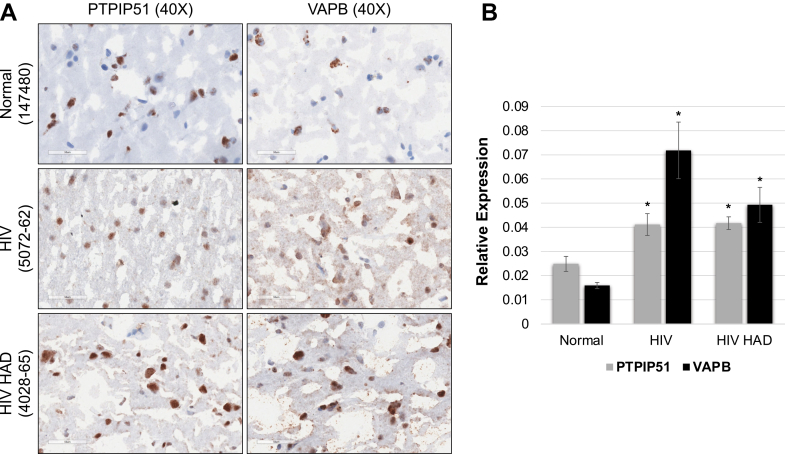
VAPB and PTPIP51 expression in HIV-infected human brains. (A) Representative immunohistochemistry of postmortem frontal cortex from normal controls (n = 3), HIV + individuals without dementia (n = 3), and HIV + individuals with HIV-associated dementia (HAD, n = 3). *Top row*: PTPIP51 staining. *Bottom row*: VAPB staining. Immunoreactivity appears brown (DAB chromogen) with hematoxylin nuclear counterstain (*blue*). Magnification 400× (40× objective with 10× ocular). Note progressive increase in VAPB staining intensity across disease stages and transition from discrete punctate PTPIP51 (Normal) to diffuse cytoplasmic distribution (HIV+, HAD). (B) Quantification of relative protein expression as a percentage of positive staining area (ImageJ color deconvolution). VAPB (*orange bars*) shows a significant progressive increase (Normal < HIV+ < HAD; ∗*P* < 0.05 vs Normal, one-way ANOVA with Tukey post-hoc). PTPIP51 (*blue bars*) shows a similar trend (∗*P* < 0.05 vs Normal). Data are mean ± SEM from three individuals per group. The paradoxical correlation between elevated VAPB and worsening cognitive outcomes indicates a failure of compensatory upregulation, whereby increased protein levels cannot restore functional tethering.

We previously established that Tat promotes PTPIP51 tyrosine phosphorylation, preventing PTPIP51-VAPB binding and depleting both proteins from MAM fractions (11). To determine whether this functional loss is recapitulated in human HIV infection, we analyzed the postmortem frontal cortex. Immunohistochemical analysis revealed significantly elevated VAPB expression in HIV-infected tissue, highest in HIV-associated dementia cases (Fig. 1A, B, *P* < 0.05), with PTPIP51 showing a similar trend. VAPB transitioned from light diffuse to intense perinuclear/reticular staining, most pronounced in HIV-associated dementia, while PTPIP51 shifted from discrete punctate to diffuse cytoplasmic distribution. The paradoxical correlation between elevated VAPB and worsening cognitive outcomes reflects a compensatory transcriptional response that is mechanistically insufficient: Tat-induced phosphorylation of PTPIP51 sterically prevents PTPIP51-VAPB binding regardless of VAPB expression level (11). This expression-function dissociation parallels that observed in ALS and Alzheimer's disease models (14, 15, 16, 17).

### Comprehensive lipidomics reveals triglyceride dysregulation

Next, we sought to determine the mechanism by which Tat induces the dissociation of VAPB-PTPIP51. Consistent with our published findings (11), phosphorylation of PTPIP51 prevents its functional interaction with VAPB. Notably, dissociated VAPB relocalizes from the perinuclear/ER region to a more diffuse cytoplasmic distribution, as shown in Fig. 1A and in our previous studies of hippocampal dentate gyrus tissues from HIV-Tg rats (11). Based on the literature, relocalization of VAPB impairs triglyceride breakdown, leading to the accumulation of lipid droplets. (31).

**Fig. 2:**
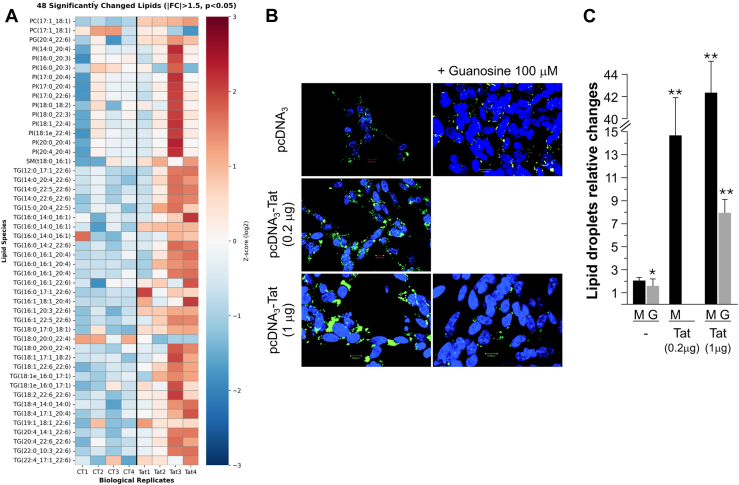
Comprehensive lipid dysregulation and dose-dependent accumulation. (A) Representative heatmap of 48 significantly altered lipid species detected by LC-MS/MS in neurons treated with Tat (100 ng/ml, 24h) versus control (fold change >1.5, *P* < 0.05) (n = 4 biological replicates). The 48 lipids displayed across classes: Triglycerides (TG; 26 species, 54%) include polyunsaturated species TG(18:0_20:0_22:4), TG(16:0_16:1_22:6), TG(18:2_22:6_22:6) containing arachidonic acid (20:4) and docosahexaenoic acid 22:6 (DHA). Phosphatidylinositols (PI; 10 species, 21%), sphingomyelins (SM), and ceramides (Cer). *Bars* represent z-score (log2 fold change): *red* = increased, *blue* = decreased. Biological replicates: CT1–4 (control), Tat1–4 (Tat-treated). ∗*P* < 0.05, ∗∗*P* < 0.01. Triglycerides comprised 54% of the altered species, dominated by polyunsaturated forms, indicating membrane catabolism rather than de novo lipogenesis. (B) Representative confocal microscopy showing lipid droplet accumulation (BODIPY 493/503, *green*) and nuclei (DAPI, *blue*) in SH-SY5Y cells transfected with empty vector (pcDNA3) or Tat-expressing constructs (0.2 or 1 μg pcDNA3-Tat) ± 100 μM guanosine. *Left* column: without guanosine. *Right* column: with guanosine. *Scale bars* represent 10 μm. Dose-response with recombinant Tat protein in supplemental Fig. S1. (C) Quantification of lipid droplet parameters from automated ImageJ analysis (>50 cells per condition, three independent experiments). One-way ANOVA with Tukey's test. ∗∗*P* < 0.01, ∗*P* < 0.05 versus pcDNA3 control; ∗∗*P* < 0.01 for Tat 1 μg + guanosine versus Tat 1 μg alone. Guanosine supplementation reduced accumulation by 68%–82%, demonstrating metabolic rescue through bioenergetic support.

To confirm this observation, we conducted lipidomic analysis. LC-MS/MS profiling of 1,796 lipid species identified 48 that were significantly altered in Tat-treated neurons (fold change >1.5, *P* < 0.05). Triglycerides made up 26 of these 48 species (54% of the response, Fig. 2A). The largest changes in polyunsaturated triglycerides included TG(18:0_20:0_22:4), TG(16:0_16:1_20:4), TG(16:0_16:1_22:6), and TG(18:2_22:6_22:6), which contain arachidonic acid (20:4) and docosahexaenoic acid (22:6) (32, 33, 34, 35) Under normal conditions, these fatty acids are mainly incorporated into membrane phospholipids or undergo mitochondrial β-oxidation (26, 36). Their buildup in storage triglycerides suggests disrupted trafficking and compromised oxidative capacity. Although the lipidomic profiles align with impaired fatty acid oxidation and phospholipid synthesis, we did not perform direct functional tests of fatty acid oxidation flux. Therefore, the proposed mechanism is supported by multiple indirect sources of evidence.

Phosphatidylinositols (10 species, 21%) and sphingolipids showed significant alterations. Sphingolipid synthesis occurs at MAMs, requiring close apposition of organelles (13, 37). Ceramide accumulation activates apoptotic pathways and promotes α-synuclein oligomerization (38, 39) (MS- unpublished data), while sphingomyelin composition determines Golgi organization.

### Microscopy confirms dose-dependent lipid droplet accumulation

Following lipid alteration, we assess the status of lipid droplets. BODIPY 493/503 staining showed dose-dependent buildup of lipid droplets (Fig. 2B, supplemental Figl S1). Control cells had few droplets; cells treated with 200–1,000 ng/ml Tat showed increasing numbers and sizes of droplets. Guanosine supplementation (100 μM) significantly reduced lipid load even at high Tat doses (Fig. 2B, C, *P* < 0.01), (40) suggesting a link to bioenergetic failure that warrants further investigation.

### VAPB silencing phenocopies Tat while PTPIP51 silencing does not

**Fig. 3:**
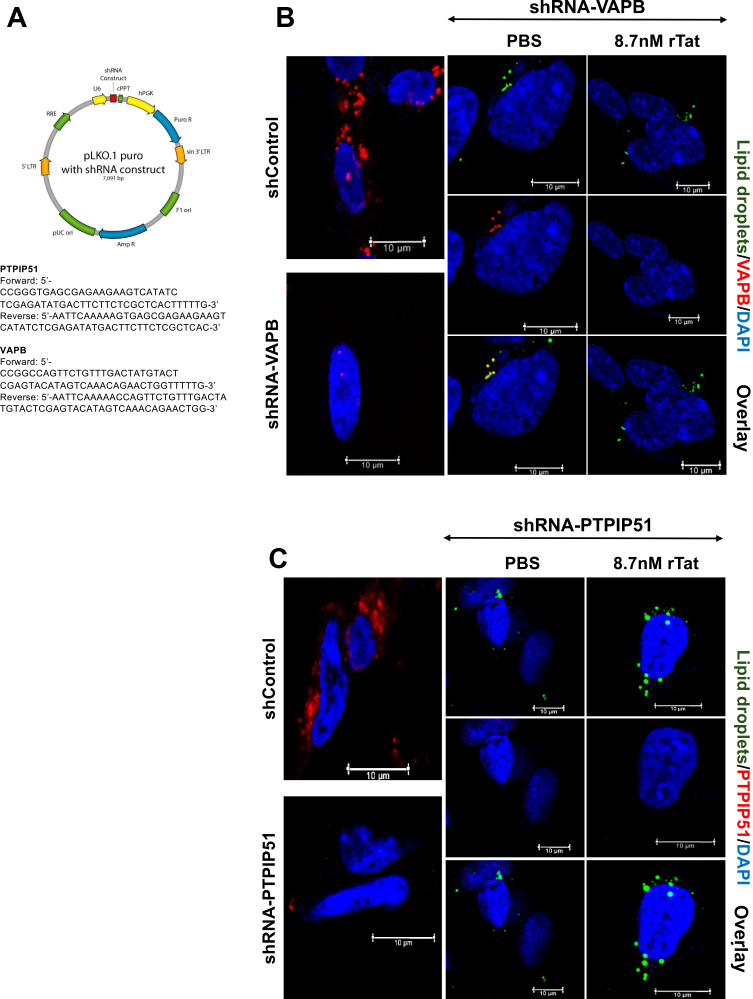
VAPB knockdown phenocopies tat-induced lipid accumulation. (A) shRNA target sequences for PTPIP51 and VAPB knockdown were cloned into pLKO.1 lentiviral vector. (B) Confocal microscopy showing lipid droplets (BODIPY 493/503, *green*), VAPB immunofluorescence (*red*), and DAPI (*blue*) with merged overlay. Column 1 (*top left*): shControl cells show baseline VAPB expression. Column 1 (*bottom left*): shRNA-VAPB cells exhibit reduced VAPB expression. Columns 2 and 3: shRNA-VAPB cells treated with PBS or Tat (8.7 nM, ∼100 ng/ml) maintain VAPB knockdown, with minimum/no further increase in lipid droplet accumulation. Scale bars represent 10 μm. (C) Confocal microscopy showing lipid droplets (*green*), PTPIP51 immunofluorescence (*red*), and DAPI (*blue*) with merged overlay. Column 1 (*top left*): shControl cells show baseline PTPIP51 expression. Column 1 (*bottom left*): shRNA-PTPIP51 cells exhibit almost total PTPIP51 expression. Columns 2 and 3: shRNA-PTPIP51 cells treated with PBS or Tat (8.7 nM, ∼100 ng/ml) maintain PTPIP51 knockdown and demonstrate that PTPIP51 loss does NOT prevent Tat-induced lipid toxicity. Scale bars represent 10 μm. This validation establishes VAPB, not PTPIP51, as the critical mechanistic node for neuronal lipid homeostasis.

To establish VAPB as the critical node, we performed shRNA-mediated loss-of-function analysis. shRNA-VAPB cells showed baseline lipid droplet accumulation. Critically, Tat treatment of shRNA-VAPB cells failed to induce further lipid droplet accumulation, establishing that VAPB is the determinant of lipid droplet formation (Fig. 3B) (14, 16). This convergence indicates that Tat drives lipid accumulation primarily through VAPB.

In contrast, PTPIP51 silencing produced minimal baseline change, and Tat treatment of PTPIP51-depleted cells resulted in full accumulation comparable to Tat-treated controls (Fig. 3C), demonstrating that PTPIP51 loss neither phenocopies nor prevents Tat-induced lipid accumulation. Taken together, three converging observations establish VAPB as the obligate mechanistic node: (1) VAPB depletion phenocopies Tat; (2) VAPB depletion occludes any additional Tat effect; (3) PTPIP51 depletion does neither.

### VAPB disruption alters ER-Golgi contacts and induces Golgi dispersal

**Fig. 4:**
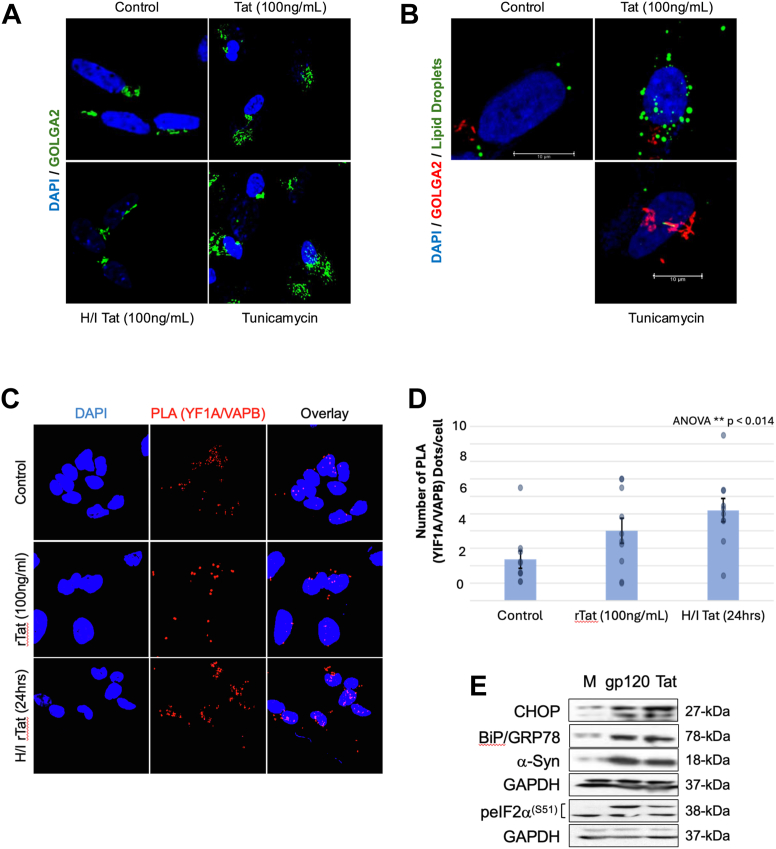
Tat disrupts VAPB-dependent contacts and triggers ER stress. (A) Immunofluorescence showing Golgi morphology visualized with anti-GOLGA2/GM130 (*green*) and DAPI (blue). Control (top left): Compact perinuclear Golgi ribbon. Tat 100 ng/ml, 24h (top right): Golgi dispersal comparable to tunicamycin with dispersed punctate structures. Heat-inactivated Tat (*bottom right*): Reduced disruption. Tunicamycin 5 μg/ml (*bottom right)*: Positive control showing induced fragmentation. Scale bars represent 10 μm. One-way ANOVA, ∗∗*P* < 0.014 for Tat versus control. Heat-inactivated Tat shows no significant reduction. (B) Using similar conditions as in (A), immunofluorescence showing Golgi morphology visualized with anti-GOLGA2/GM130 (*red*), lipid droplets (*green*), and DAPI (*blue*). Control (*top left*): Compact perinuclear Golgi ribbon and low lipid droplet accumulation. Tat (*top right*): Lipid droplet accumulation and Golgi dispersal that is masked by LD. Tunicamycin 5 μg/ml (bottom right): Positive control showing induced fragmentation with no visible LD. Scale bars represent 10 μm. One-way ANOVA, ∗∗*P* < 0.05 for Tat versus control. (C) Recombinant Tat 100 ng/ml, 24h (*right*): Modest nonsignificant increase in PLA dots per cell, consistent with aberrant complex clustering or conformational disruption rather than simple quantitative reduction of interaction. Heat-inactivated Tat (*middle*): Statistically significant increase in PLA signal (*P* < 0.014), consistent with compensatory interaction when Tat's disruptive activity is absent. (D) Quantification of YIF1A–VAPB interaction showing the number of PLA dots per cell from >50 cells per condition across three independent experiments. Bars show mean ± SEM. (E) Western blot analysis showing ER stress markers in differentiated SH-SY5Y neurons treated with control (CT), gp120, or Tat (100 ng/ml, 24 h). Markers: CHOP (27 kDa), BiP/GRP78 (78 kDa), phosphorylated eIF2α(S51) (38 kDa), total eIF2α (38 kDa), α-synuclein (18 kDa), GAPDH loading control (37 kDa). Representative of two to three independent experiments.

As mentioned above, altered lipid levels determine Golgi status; therefore, we sought to examine Golgi organization in Tat-treated differentiated SH-SY5Y cells. Immunofluorescence with anti-GM130/Golga2 revealed progressive Golgi dispersal in Tat-treated cells at 48 h (Fig. 4A). In control cells, GM130 immunoreactivity was concentrated in compact, ribbon-like perinuclear structures consistent with intact Golgi architecture. Following Tat treatment, GM130-positive structures displayed pronounced dispersal from the perinuclear region, with loss of cisternal continuity, the appearance of dispersed perinuclear structures, and redistribution across a broader cytoplasmic area, which persisted at 48 h. Heat-inactivated Tat produced an intermediate phenotype, with largely preserved perinuclear ribbon organization but occasional small discrete puncta, demonstrating that full Golgi dispersal requires active Tat protein conformation. Tunicamycin served as a positive control for complete Golgi fragmentation, a morphologically distinct phenotype characterized by vesiculated puncta dispersed throughout the cytoplasm, clearly distinguishable from the perinuclear dispersal induced by Tat.

Using the same approach, we examined the levels of lipid droplets and golgi in these cells. Differentiated SH-SY5Y cells were treated with Tat for 24 h, followed by tunicamycin treatment for 4 h. As shown in Fig. 4B, the addition of Tat, but not tunicamycin, increases lipid droplet accumulation. These results are normal since tunicamycin was not confirmed to affect lipid droplets in neurons as in hepatocytes. Interestingly, while the levels of fragmented Golgi increase in tunicamycin-treated neurons, the apparent discrepancy in Tat-treated cells seems to be more about spatial redistribution/masking in double staining, rather than a lack of Golgi induction.

Further, VAPB coordinates ER contacts with multiple organelles, not just mitochondria, and loss of its function prevents this connection (13, 16). Hence, we investigated this association in Tat-treated differentiated SH-SY5Y cells. Proximity ligation assay revealed that Tat induced a modest increase in YIF1A-VAPB interaction dots per cell, while heat-inactivated Tat produced a significant increase (*P* < 0.014, Fig. 4C, D). This paradoxical pattern is consistent with active Tat disrupting the functional conformation of the YIF1A-VAPB complex while promoting aberrant clustering, whereas heat-inactivated Tat, lacking this disruptive activity, allows compensatory interaction (13).

Finally, we characterized the cellular consequences of VAPB disruption on the ER. We treated differentiated SH-SY5Y neurons with recombinant Tat protein (100 ng/ml, 24h). Western blot analysis demonstrated induction of ER stress markers: CHOP increased, BiP/GRP78 increased, and eIF2α phosphorylation increased (Fig. 4E) (41). BiP/GRP78 is upregulated during ER stress to bind and stabilize misfolded proteins; however, when misfolded protein load exceeds its buffering capacity, BiP becomes saturated, and proteostasis cannot be maintained. These findings establish that VAPB disruption is associated with ER dysfunction beyond MAM-specific effects. However, it also means that Tat might be using two separate mechanisms to induce ER stress and lipid droplet accumulation.

### Metabolomic profiling reveals bioenergetic failure

**Fig. 5:**
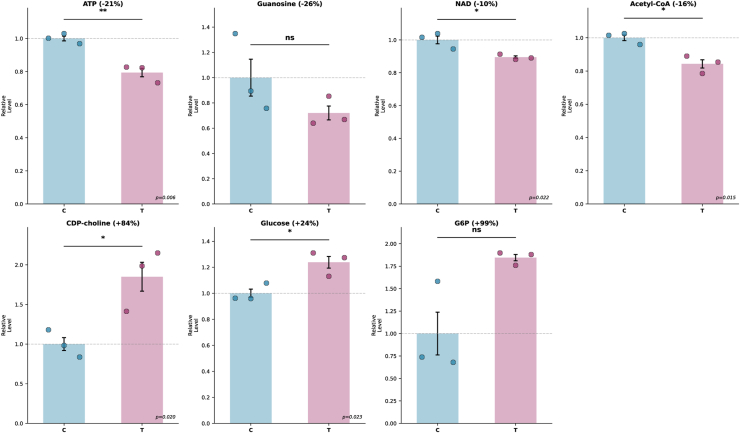
Metabolomic profiling reveals bioenergetic failure. Targeted metabolomic analysis of SH-SY5Y neurons treated with Tat (100 ng/ml, 24 h) identified seven key metabolites with altered abundance in energy metabolism and phospholipid synthesis pathways. Bar graphs show mean ± SEM with individual biological replicates as dots (n = 3 per group). Values normalized to control mean (*dashed line* = 1.0). *Orange* = Control, *Blue* = Tat. Energy metabolites: ATP decreased 21% (*P* = 0.006, ∗∗), confirming bioenergetic failure that impairs fatty acid β-oxidation; guanosine (ns), mechanistically explaining why guanosine supplementation rescued lipid accumulation; NAD decreased 10% (*P* = 0.022, ∗); acetyl-CoA decreased 16% (*P* = 0.015, ∗), confirming impaired oxidative metabolism. Phospholipid synthesis bottleneck: CDP-choline (the rate-limiting precursor for phosphatidylcholine synthesis) increased by 84% (*P* = 0.020, ∗∗), indicating that substrate accumulates while downstream biosynthesis is blocked, forcing lipid diversion from membrane synthesis to neutral storage. Glycolytic dysfunction: Glucose increased 24% (*P* = 0.023, ∗); Glucose-6-phosphate (ns), demonstrating "metabolic mismatch" where substrate accumulates but cannot be efficiently metabolized. ∗*P* < 0.05, ∗∗*P* < 0.01, ns = not significant. Complete metabolomics deposited to NIH Metabolomics Workbench.

To understand the mechanistic basis for lipid accumulation, we performed targeted metabolomics. Mass spectrometry identified 10 significantly altered metabolites clustered in energy metabolism and phospholipid synthesis pathways (Fig. 5).

Energy metabolites were profoundly depleted: ATP decreased 21% (*P* = 0.006), NAD decreased 10% (*P* = 0.022), and acetyl-CoA decreased 16% (*P* = 0.015) (42, 43, 44). These three metabolites together constitute a bioenergetic failure signature consistent with impaired mitochondrial oxidative function, corroborating the ROS accumulation and mitochondrial stress documented in the same cell model in Arjona *et al.* 2023 (11). Acetyl-CoA depletion is particularly informative as the obligate end-product of fatty acid β-oxidation: its reduction in the context of triglyceride accumulation is mechanistically consistent with, though not direct proof of, impaired oxidative flux. CDP-choline increased 84% (*P* = 0.020), revealing a critical bottleneck: (42) this rate-limiting precursor for phosphatidylcholine synthesis accumulates when downstream biosynthesis is blocked, consistent with MAM dysfunction (37, 45).

Guanosine showed a trend toward decrease, and glucose-6-phosphate showed a trend toward increase, consistent with metabolic mismatch, though these did not reach statistical significance due to high biological variability in baseline nucleotide and glycolytic pools (*P* > 0.05, Fig. 5). The core bioenergetic failure conclusion, therefore, rests on the three statistically significant metabolites above.

The CDP-choline accumulation explains triglyceride accumulation: cells divert lipids from membrane synthesis (blocked at MAMs) to neutral storage (functional), explaining triglyceride accumulation despite impaired phospholipid production (42). Glycolytic intermediates showed trends consistent with “metabolic mismatch,” in which substrate accumulates but cannot be efficiently metabolized, (43, 44) though individual glycolytic metabolite changes did not all reach statistical significance.

## Discussion

### VAPB as master regulator of organellar homeostasis

This study establishes VAPB as a master regulator whose disruption initiates a cascade of organellar failures in a differentiated neuronal cell model. While widely used, SH-SY5Y cells still have limitations and may not fully recapitulate the characteristics of terminally differentiated primary neurons (45). All conclusions are therefore framed as findings in this model system, validated where possible by human postmortem data. Future studies will include primary neurons and iPSCs (induced Pluripotent Stem cells). The sequence we document, PTPIP51 phosphorylation, dissociates VAPB from PTPIP51 and relocates VAPB from the ER/nucleus to the cytoplasm. In return, VAPB loss-of-function impairs lipid levels, leading to impaired organelle contacts (ER-mitochondria, ER-Golgi), thereby causing proteostatic failure and metabolic collapse, positioning VAPB loss as the upstream trigger (17, 37, 46, 47).

Knockdown analysis demonstrates that VAPB itself is the critical node. VAPB knockdown phenocopied the entire cascade; PTPIP51 knockdown did not. VAPB coordinates a multi-organellar network whose collapse creates the pathological phenotype.

### The lipid profile indicates membrane catabolism

The polyunsaturated triglyceride profile indicates membrane phospholipid breakdown rather than de novo lipogenesis (18, 19, 20, 23). Specifically, accumulation of TG species containing arachidonic acid and docosahexaenoic acid, the two most abundant polyunsaturated fatty acids in neuronal membranes, suggests membrane catabolism (33, 34, 35, 36). Multiple lines of evidence support this: *1-* polyunsaturated TG species derive from membrane precursors; *2-* guanosine supplementation rescued accumulation potentially by providing ATP for β-oxidation (40); *3-* VAPB knockdown phenocopied accumulation, implicating contact site-dependent trafficking (16); *4-* concordant CDP-choline and phosphatidylcholine perturbations indicate impaired phospholipid biosynthesis (42).

### Organellar dysfunction converges on lipid pathology

Multiple failures converge to drive lipid accumulation and organelle deregulation. It has been shown that VAPB dissociation from oxysterol-binding protein-related protein 2 and ESYT1/2 can trigger this phenomenon (48, 49, 50). From MAMs: impaired Ca^2+^-stimulated oxidative metabolism reduces fatty acid β-oxidation, as evidenced by acetyl-CoA depletion (26, 37, 46). From Golgi: dispersed Golgi cannot properly synthesize or traffic membrane lipids, forcing diversion to storage. CDP-choline accumulation despite Golgi dysfunction suggests a synthesis bottleneck in which precursors accumulate, but products cannot form (42). From energy failure: ATP depletion (21%) prevents both lipid catabolism and trafficking (43, 44). Lipid droplets initially represent adaptive storage, but excessive accumulation signals complete metabolic failure (22, 25).

We emphasize that while the convergence of lipidomic and morphological data is consistent with impaired fatty acid oxidation and phospholipid trafficking, direct functional assays of fatty acid oxidation flux, lipid transfer rates, or phospholipid synthesis were not performed in this study. These constitute important future experiments that would further strengthen the mechanistic model.

### ER stress amplifies the initial disruption

ER stress acts as both a consequence and an amplifier (41). VAPB loss triggers ER stress (peIF2α, BiP/GRP78, CHOP), which then impairs additional VAPB-dependent functions (14, 15, 41). ER stress disrupts COPII-mediated ER-Golgi trafficking, explaining Golgi fragmentation/dispersal (13). ER stress overwhelms the UPR, explaining α-synuclein accumulation (38, 41, 51, 52). This creates positive feedback amplifying the initial VAPB disruption. However, our data show that the use of small-interfering RNA directed against BiP/GRP78 prevents Tat from altering the expression levels of CHOP, BiP/GRP78, and peIF2a, indicating that Tat uses a different mechanism to directly induce ER stress, which could involve the Cav1.2 protein (MS- unpublished data).

Metabolic and Proteostatic Failure ConvergeLipid droplet and α-synuclein colocalization is not merely correlative. (25, 38, 39, 41, 51, 52) Lipid droplets seed α-synuclein aggregation by providing hydrophobic surfaces, while α-synuclein recruits lipid droplets through amphipathic helix interactions (39). This creates self-amplifying metabolic-proteostatic failure (38, 51). Ceramide-enriched membranes enhance α-synuclein binding (35). Phosphatidylinositol dysregulation, particularly PI_3_P, essential for autophagosome formation, implicates impaired autophagy (53).

### Potential implications for cerebrovascular risk

People living with HIV experience a 1.5-2-fold elevated stroke risk despite viral suppression (1, 2, 3, 4, 5, 54, 55, 56). The lipid profile we document, polyunsaturated triglycerides, depleted plasmalogens, and elevated ceramides, is mechanistically implicated in ischemic pathology (18, 19, 20, 23, 57, 58, 59). We emphasize that these parallels are observational and speculative. We do not claim causal evidence for stroke susceptibility and acknowledge that direct demonstration would require HIV + stroke tissue and animal models combining chronic Tat exposure with experimental cerebral ischemia, approaches that are beyond the scope of the current study. The comparison is offered as a plausible mechanistic hypothesis warranting future investigation (supplemental Table S1) (1, 54, 60, 61).

### Therapeutic implications

VAPB emerges as a master regulator whose stabilization could prevent this cascade. Strategies that merely increase VAPB expression are unlikely to succeed, as demonstrated by the paradoxical correlation between elevated VAPB and worse outcomes (16, 17). Effective interventions must restore functional PTPIP51 localization or stabilize contacts through PTPIP51-independent mechanisms. Kinase inhibitors that prevent PTPIP51 phosphorylation (dasatinib, gefitinib) constitute a rational approach (16). Bioenergetic support with guanosine is particularly attractive given the dramatic rescue observed (40). Guanosine and uridine have acceptable safety profiles in clinical trials for neurological disorders (40).

### Limitations

We acknowledge the following limitations of this study, which define the boundaries of interpretation and identify directions for future work.

First, we employed SH-SY5Y neuroblastoma cells rather than primary terminally differentiated neurons. While retinoic acid differentiation induces neuronal morphology, marker expression, and electrophysiological properties, tumor-derived cell lines behave differently from primary neurons in important respects. The translational relevance of our findings is supported by two layers of human validation: (1) VAPB dysregulation documented in Arjona *et al.* 2023 was validated in human postmortem brain tissue (11); and (2) the current study adds VAPB expression correlating with dementia severity in HIV-infected frontal cortex. Primary neuron validation, in rat hippocampal neurons and human iPSC-derived neurons, is identified as the priority next experimental step. We believe the convergence of in vitro, multiomics, and human translational evidence justifies reporting these findings, while explicitly acknowledging this limitation.

Second, we did not perform direct fatty acid oxidation flux assays (e.g., Seahorse FAO stress test) or lipid trafficking assays (e.g., BODIPY C12 pulse-chase). Our evidence for impaired β-oxidation is indirect, relying on acetyl-CoA depletion as a surrogate endpoint, pharmacological rescue with guanosine, and the accumulation profile of polyunsaturated fatty acids (supplemental Table S1). These represent mechanistically coherent but indirect lines of evidence. Direct flux assays are identified as a future experimental direction pending restoration of laboratory resources.

Third, human tissue immunohistochemistry demonstrates altered VAPB expression but cannot establish contact site functional integrity at standard resolution. Super-resolution microscopy or proximity ligation assay on frozen tissue sections would be required for functional validation in human tissue.

Fourth, we demonstrate colocalization of lipid droplets and α-synuclein but did not establish causal directionality between these two pathologies.

Fifth, the cerebrovascular risk implications remain speculative and require animal models that combine chronic Tat exposure with experimental cerebral ischemia (supplemental Table S1).

Despite these limitations, the convergence of shRNA-mediated loss-of-function evidence, pharmacological rescue, multiomics profiling, and human translational data provides mechanistically coherent support for the central finding that VAPB disruption is associated with a cascade of organellar and metabolic failure in HIV neuropathogenesis (62).

## Conclusion

HIV-1 Tat disrupts VAPB-dependent organellar contacts in differentiated SH-SY5Y neurons, initiating a cascade of ER stress, Golgi dispersal, protein aggregation, lipid accumulation, and bioenergetic failure. VAPB knockdown phenocopies this cascade while PTPIP51 knockdown does not, establishing VAPB as the critical mechanistic node. VAPB expression is elevated in HIV-infected human brains, correlating with dementia severity, revealing a pattern consistent with failed compensatory upregulation. These findings, generated in a well-characterized neuronal model and corroborated by human tissue data, identify VAPB disruption as a unifying mechanism in HIV neuropathogenesis and position VAPB-dependent contact site integrity as a rational therapeutic target.

## Data availability

Raw lipidomics and metabolomics data, image files, and analysis scripts are available from the corresponding author upon reasonable request.

## Supplemental data

This article contains supplemental data (63, 64, 65, 66, 67, 68, 69, 70, 71, 72).

## Declaration of Generative AI and AI-Assisted Technologies in the Writing Process

During the preparation of this work, the authors used ***Grammarly*** for clarity and readability. After using this tool/service, the author(s) reviewed and edited the content as needed and take(s) full responsibility for the content of the published article.

## Conflicts of interest

The authors declare no conflicts of interest.
