# Supplementary figures and images for: HIV-1 Tat-induced VAPB disruption initiates a cascade of organellar failures culminating in neuronal lipid accumulation
Source: J Lipid Res. 2026 May 4;67(6):101053. doi: 10.1016/j.jlr.2026.101053 (PMC13234481; doi:10.1016/j.jlr.2026.101053)

FIGURE 1S

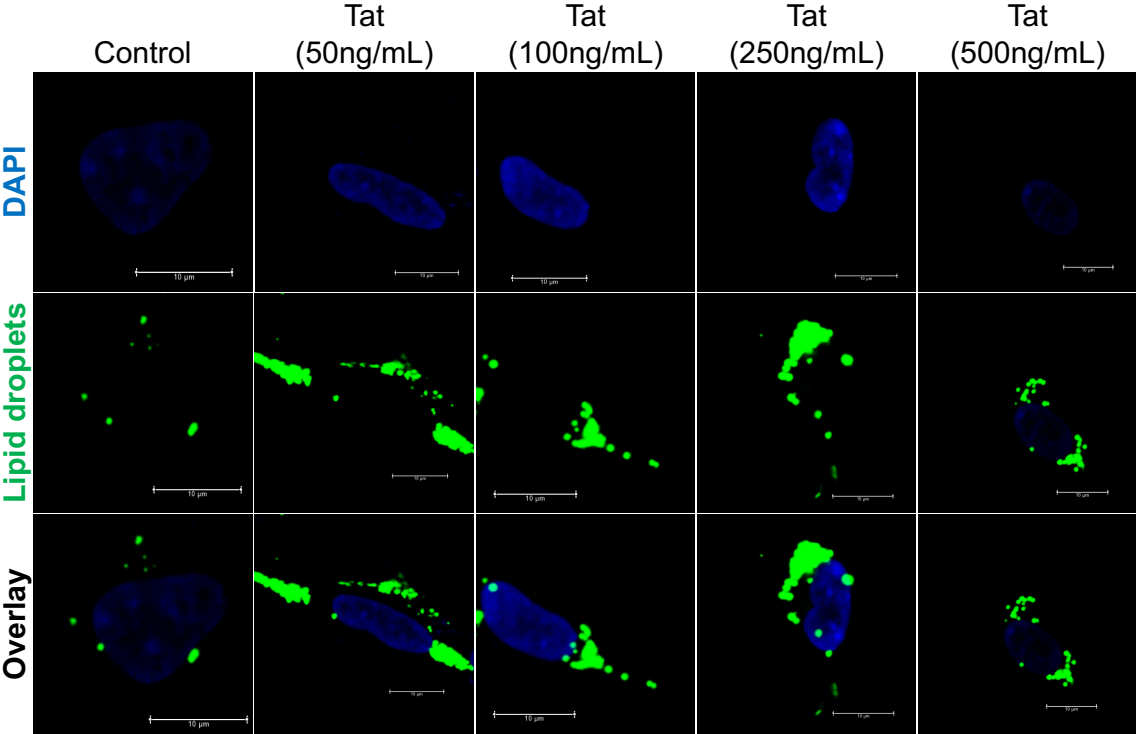

TABLE S1

Supplement: Supporting Figure S1 [file mmc1.pdf]
