# Supplementary material for: HIV-1 Tat-induced VAPB disruption initiates a cascade of organellar failures culminating in neuronal lipid accumulation
Source: J Lipid Res. 2026 May 4;67(6):101053. doi: 10.1016/j.jlr.2026.101053 (PMC13234481; doi:10.1016/j.jlr.2026.101053)
Supplement: Supporting Table S1 [file mmc2.pdf]

Clinical Observation	Key Finding	Population	References
Hypertriglyceridemia prevalence in PLWH on ART	40-66% of patients on PI-based HAART develop elevated triglycerides (>150 mg/dL)	Multicenter cohorts, USA/Europe	63
Triglyceride-glucose (TyG) index and CVD risk	Each 1-SD increase in TyG index associated with 39% higher CVD risk (HR 1.39, 95% CI 1.22-1.59); high-increasing trajectory = 2.92-fold increased CVD risk	16,122 treatment-naive PLWH, China, 2005-2022	64
Stroke incidence in PLWH	PLWH exhibit 2-fold higher CVD incidence vs HIV-negative; stroke hospitalizations increased 60% from 1997-2006 in PLWH while declining 7% in general population	US national datasets	65 and ref within
Hypertriglyceridemia as ischemic stroke risk factor	Elevated non-fasting triglycerides correlated with ischemic stroke risk via atherosclerosis, thrombosis, and increased blood viscosity	General population + HIV cohorts	56, 66
Lipid dysregulation in HAND	Progressive impairments in energy and lipid metabolism worsen with age and HIV duration; bioactive lipids (ceramide, sphingomyelin) accumulate in HAND brains	HIV+ individuals with ANI/MND	67, 68
ART-independent neuronal lipid accumulation	Brain lipid/energy metabolic changes present even with viral suppression on cART; changes in bioenergetics measurable by MRS in early cognitive impairment	Longitudinal HIV+ cohorts	69, 70
Stroke in PLWH - younger age of onset	PLWH with stroke significantly younger (median 46.5 yrs) vs HIV-negative stroke patients (median 61.0 yrs); 83.3% had dyslipidemia	Case-control study, West Africa (n=540)	71
IRIS-associated stroke	Almost 1/3 of strokes in PLWH may be related to IRIS; crude incidence rate 3.5x higher in first 6 months post-ART initiation	77 PLWH with new-onset stroke, South Africa	72
