# Supplementary material for: HIV-1 Tat-induced VAPB disruption initiates a cascade of organellar failures culminating in neuronal lipid accumulation
Source: J Lipid Res. 2026 May 4;67(6):101053. doi: 10.1016/j.jlr.2026.101053 (PMC13234481; doi:10.1016/j.jlr.2026.101053)
Supplement: Supporting information [file mmc3.docx]

**SUPPORTING INFORMATION**

**TABLE S1. Clinical Evidence Linking HIV Infection, Hypertriglyceridemia, and Cerebrovascular Risk.** Abbreviations: PLWH = People living with HIV; ART = antiretroviral therapy; PI = protease inhibitor; HAART = highly active antiretroviral therapy; HAND = HIV-associated neurocognitive disorder; TyG = triglyceride-glucose; CVD = cardiovascular disease; ANI = asymptomatic neurocognitive impairment; MND = mild neurocognitive disorder; IRIS = immune reconstitution inflammatory syndrome; MRS = magnetic resonance spectroscopy.

**FIGURE S1. Dose-Dependent Lipid Accumulation with Recombinant Tat Protein.** Representative confocal microscopy showing lipid droplets (BODIPY 493/503, green) and nuclei (DAPI, blue) in differentiated SH-SY5Y cells treated with increasing concentrations of recombinant HIV-1 Tat protein for 24h. Top row: BODIPY channel. Bottom row: merged BODIPY + DAPI. Control (vehicle): Minimal baseline. Tat 50 ng/mL: Mild accumulation. Tat 100 ng/mL: Moderate accumulation. Tat 250 ng/mL: Substantial accumulation. Tat 500 ng/mL: Massive accumulation filling cytoplasm. Scale bars 10 μm. Validates that lipid accumulation is reproducible across Tat delivery methods (recombinant protein vs transfection) and is dose-dependent.
